# Effects of Kamishoyosan, a Traditional Japanese Medicine, on Menopausal Symptoms: A Randomized, Placebo-Controlled, Double-Blind Clinical Trial

**DOI:** 10.1155/2020/9285317

**Published:** 2020-07-11

**Authors:** Kiyoshi Takamatsu, Mariko Ogawa, Tsuyoshi Higuchi, Takashi Takeda, Kunihiko Hayashi, Hideki Mizunuma

**Affiliations:** ^1^Department of Obstetrics and Gynecology, Tokyo Dental College Ichikawa General Hospital, Sugano 5-11-13, Ichikawa-City, Chiba 272-8513, Japan; ^2^Department of Nursing Science, Hirosaki University Graduate School of Health Sciences, Zaifu-cho 5, Hirosaki-City, Aomori 036-8562, Japan; ^3^Division of Women's Health, Research Institute of Traditional Asian Medicine, Kindai University, Oonohigashi 377-2, Osaka-sayama-City, Osaka 589-8511, Japan; ^4^Department of International/Community Health Laboratory Sciences, Graduate School of Health Sciences, Gunma University, Showa-Machi 3-39-22, Maebashi-city, Gunma 371-8514, Japan; ^5^Fukushima Medical Center for Children and Women, Fukushima Medical University, Hikariga-oka 1, Fukushima-City, Fukushima 960-1295, Japan

## Abstract

**Objective:**

Kampo medicine, a traditional Japanese medicine, is widely used in Japan, especially in the field of menopause medicine. However, few studies have shown evidence-based effects. This study aimed to confirm the effects of kamishoyosan on menopausal symptoms with a randomized, placebo-controlled, double-blind clinical trial.

**Methods:**

Subjects were randomly allocated to groups that received either kamishoyosan (*n* = 101) or a placebo resembling kamishoyosan (*n* = 104). The primary outcomes were the change in the number of hot flashes, depression scores, improvements of anxiety, quality of life (QOL), and menopausal symptoms before and 4 and 8 weeks after initiation of treatment with the study drug. The secondary outcome was drug safety.

**Results:**

After 8 weeks, the number of hot flashes decreased after treatment in both groups, but there was no significant difference between the two groups. The changes in SDS scores showed the same results. Moreover, no significant differences were observed between the two groups in assessments with the STAI, SF-36, and JSOG menopausal index. No serious adverse effect was reported.

**Conclusions:**

This first placebo-controlled double-blind randomized trial with kamishoyosan demonstrated that it was safe and had some effects on climacteric symptoms, but not significant compared with placebo. Some problems, such as placebo effects, in the study of Kampo therapy for menopausal symptoms, were revealed. This trial is registered with the trial registration number. UMIN 000006042.

## 1. Introduction

Menopausal symptoms are characterized by a wide range of variations and have been known to significantly and negatively affect the quality of life (QOL) of women during menopause [1]. They are also associated with many issues from the medical-economic perspective, including overexamination for diagnosis and symptom treatment and prescription of inadequate medication. Moreover, besides hormonal factors, many causes, such as living environment and personality factors, affect symptoms [2]. For these reasons, various methods are employed for the treatment, including counseling and drug therapy [3].

Among drug therapies, it is well known that hormone therapy (HT) is pertinent to and has an extremely high response rate for hot flashes, sweating, and sleep disturbances. However, the effects of HT on other symptoms are somewhat limited, especially those involving the neuropsychiatric system [4]. The risk of breast cancer has been pointed out as an issue as well [5, 6]. Meanwhile, Kampo therapy, traditional Japanese medicine, has gained support from many physicians and patients for its high safety and mechanism of action. There is, however, little scientific evidence to support its effects from the view point of evidence-based medicine, which is widely employed in Western medicine [7].

Among Kampo formula, kamishoyosan is frequently prescribed to relieve various menopause-related symptoms. It reportedly improved anxiety and depression in midlife women with psychological symptoms [8] and improved sleep disturbance in Japanese peri- and postmenopausal women [9]. In basic studies, kamishoyosan was shown to exert an anxiolytic effect through stimulation of the *γ*-aminobutyric acid A (GABAA)-benzodiazepine receptor in male mice [10] and socially isolated ovariectomized rats [11]. Moreover, kamishoyosan was shown to exert antidepressive effects through 5-HT1A receptor and PKA-CREB-BDNF signaling in postmenopausal depression model mice [12].

To determine the efficacy and safety of kamishoyosan, in this study, we conducted a randomized, placebo-controlled, double-blind clinical trial with patients who had menopausal symptoms.

## 2. Subjects and Methods

### 2.1. Subjects

Among patients aged 40–60 years with a chief complaint of climacteric disorders who visited one of the 13 outpatient gynecological clinics consisting of four university hospitals and 9 related hospitals which the authors are affiliated to, we selected those with hot flashes, insomnia, headache, and/or neurological symptoms and those who were diagnosed with menopausal symptoms by the authors during the period spanning April 2011 through February 2013.

#### 2.1.1. Inclusion Criteria

Patients who suffered from the aforementioned symptoms in their daily life were recruited. The menopausal status was not taken into consideration.

#### 2.1.2. Exclusion Criteria

Patients with serious comorbidities (e.g., liver disease, kidney disease, heart disease, lung disease, hematologic disease, and malignant tumor) and a history of HT or Kampo therapy within 4 weeks prior to treatment initiation were excluded from this study. Following the informed-consent procedure, the depression status of all patients was assessed using the Zung's self-rating Depression Scale (SDS) [13]. To exclude the severely depressed patients, those who scored <62 points were finally considered eligible for this study.

### 2.2. Study Design

This study is of a multicenter, randomized, double-blind, placebo-controlled design to examine the pharmacological efficacy and safety of kamishoyosan therapy in menopausal patients at 13 sites in Japan, and is registered in UMIN Clinical Trials Registry in Japan (UMIN 000006042). A summary of this study is on UMIN Website (https://upload.umin.ac.jp/cgi-open-bin/ctr/ctr_view.cgi?recptno=R000006042). The study was conducted in accordance with the ethical guidelines for clinical studies, considering patients' human rights and privacy. The study protocol was approved by the Institutional Review Board of each institution.

### 2.3. Sample Size

The sample size was calculated for one of the primary outcomes, changes in the SDS score, and the analysis of the comparison of mean changes at 8 weeks between the kamishoyosan and placebo groups. Based on the data from the dose setting study of fluvoxamine malate (internal materials of approval review), we assumed the changes of the SDS score to be −4.7 and −2.2 in the kamishoyosan group and placebo group, respectively, with a common standard deviation of 6.0. A sample size of 92 per group was calculated to obtain a power of 80% and a level of significance of 0.05. Taking into account 10% dropout, the total sample size was set at 204.

### 2.4. Randomization

Subjects were randomly assigned to receive 7.5 g tid kamishoyosan (kamishoyosan group) or 7.5 g tid placebo (placebo group) for 8 weeks according to a computer-generated randomization list provided by a statistician from the site management organization (CRO, Sogo Rinsho Medefi Co., Ltd., Tokyo, Japan). The placebo was produced to have a similar smell and taste to kamishoyosan. Granulated powders of kamishoyosan and placebo appeared identical and were identically packaged and labeled by the CRO to maintain blinding. Subjects, investigators, and all other personnel involved in conducting the study were blinded to the treatment allocation. During the baseline period (0 weeks), subjects were assigned a randomized number. These numbers were allocated in sequential order and registered in the patient enrollment center (Sogo Rinsho Medefi Co., Ltd.). The allocation was concealed. Emergency envelopes containing the randomization code were kept under lock and key by the CRO and were examined at the end of the trial to ensure that blinding had been maintained.

### 2.5. Study Procedures and Drugs

After the 2-week pretreatment observation period, subjects initiated treatment with the study drug or placebo. Subjects were administered either kamishoyosan (Tsumura-Kampo kamishoyosan Extract Granules) or placebo having a similar appearance, smell, and taste to kamishoyosan (Tsumura & Co., Tokyo, Japan) at a dose of 7.5 g/day divided into three portions and taken before or after each meal. Kamishoyosan was used in the form of a powdered extract obtained by spray drying a hot extract mixture of the following 10 crude herbs: 13.3% Bupleuri Radix (*Bupleurum falcatum*), 13.3% Paeoniae Radix (*Paeonia lactiflora*), 13.3% Atractylodis Rhizoma (*Atractylodes ovate*), 13.3% Angelicae Radix (*Angelica acutiloba*), 13.3% Hoelen (*Poria cocos*), 8.9% Gardeniae Fructus (*Gardenia jasminoides*), 8.9% Moutan Cortex (*Paeonia suffruticosa*), 6.7% Glycyrrhizae Radix (*Glycyrrhiza uralensis*), 4.4% Zingiberis Rhizoma (*Zingiber officinale*), and 4.4% Menthae Herba (*Menthae arvensis*).

After randomization, patients were asked to visit the hospital every four weeks.

### 2.6. Concomitant Drugs/Combination Therapy

Throughout the study period, subjects were prohibited from using concomitant Kampo medicine. In addition, administration of drugs considered to affect evaluations during the study period (e.g., sex steroid hormones, anxiolytics, antidepressants, SSRIs, SNRIs, autonomic drugs, kallidinogenase preparations, glycyrrhizin-containing preparations, glycyrrhizic acid-containing preparations, black cohosh) was prohibited in general. Supplements, such as soy supplements, were allowed if the usage had been the same throughout the study period.

### 2.7. Primary and Secondary Outcomes

The primary outcome was the efficacy of kamishoyosan, the number of hot flashes, which was investigated every day using a self-reported diary and shown by the mixed effect model for repeated measures, and changes in SDS scores, anxiety assessed using the State-Trait Anxiety Inventory (STAI) [14], QOL with SF-36 [15], and menopausal symptoms with the menopausal index created by the Japan Society of Obstetrics and Gynecology (JSOG) [16], respectively, before and 4 and 8 weeks after initiation of treatment with the study drug. The JSOG menopausal index evaluates 21 symptoms with three ranks; none, mild, and severe.

To evaluate drug safety for secondary outcome, blood chemical examinations were performed, and weight and blood pressure were monitored before and after 4 weeks of treatment, as well as after treatment discontinuation.

### 2.8. Adverse Events and Safety

Safety and tolerability were assessed by recording all adverse events which were based on declarations by subjects and the result of blood sample analysis. Adverse events were defined as “adverse drug reactions” if a causal relationship with the study drug could not be ruled out, and the frequency of occurrence of adverse events and adverse drug reactions was tabulated. “Serious” adverse events were defined as (1) any event that leads to death, (2) any event that is life-threatening, (3) any event that requires inpatient hospitalization or prolongation of existing hospitalization for treatment, (4) any event that results in persistent or significant disability/incapacity, and (5) any event that results in congenital anomaly or birth defect.

### 2.9. Statistical Analysis

In consideration of missing data, changes in the number of hot flashes were calculated by a mixed effect model using values described in patient diaries in the Full Analysis Set. For analyzing the number of hot flashes, the logarithmic conversion was employed because it showed a skewed distribution. When the number was 0, it was calculated as log (number + 1). For comparing the changes over time, a repeated-measures analysis of variance for changes from the baseline value was performed with the group as the factor (i.e., kamishoyosan group or placebo group), results before initiation of treatment as covariates, and results after completion of treatment as dependent variables. Also, analysis with ANOVA was done for sensitivity analysis. The analysis of the SDS score, STAI, and SF-36 was performed with ANOVA. Summary statistics were calculated for differences in results before and after initiation of treatment as well as rates of change between the two groups. Comparisons between the kamishoyosan and placebo groups were performed using the *t*-test.

For safety evaluation, the frequency and proportion of subjects who developed any adverse events or adverse drug reactions in the kamishoyosan and placebo groups were determined along with 95% confidence intervals (CIs) and examined using Fisher's exact test.

Analyses were carried out using SAS 9.2 software (SAS Institute Inc., NC, USA). The significance level was set at 0.05.

## 3. Results

### 3.1. Participant Selection

**Figure 1:**
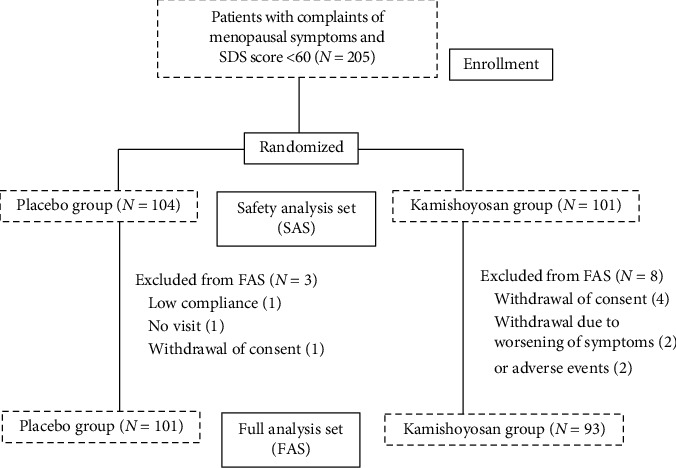
The flow of participant selection.

Among registered patients who provided consent, the 205 who scored <62 on the SDS assessment were enrolled as subjects during the trial period. Of these 205 subjects, 101 were randomly allocated to the kamishoyosan group and the remaining 104 to the placebo group (Safety Analysis Set, SAS). There were 11 who subjects discontinued treatment for reasons such as low compliance (1 subject), no visit (1 subject), withdrawal of consent (5 subjects), withdrawal due to worsening of symptoms (2 subjects), and adverse drug reactions (2 subjects). Accordingly, the full analysis set (Full Analysis Set; FAS) for efficacy assessments consisted of 194 subjects (kamishoyosan group, 93; placebo group, 101) (Figure 1).

### 3.2. Subject Characteristics

Subject characteristics for each group are shown in Table 1. We did not find differences between the groups in all inspection items.

### 3.3. Efficacy of Kamishoyosan

**Figure 2:**
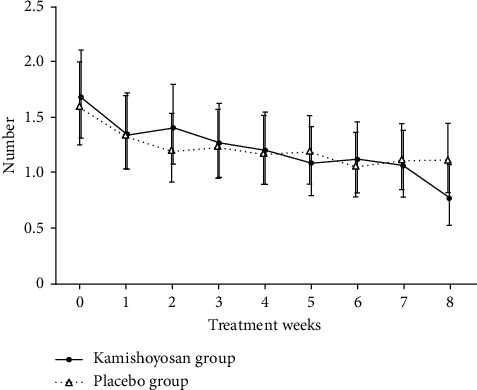
Changes in the number of hot flashes. The mean number of hot flashes was calculated using a mixed effect model based on values described in patient diaries. The number was logarithmically converted. When the number was 0, it was calculated as log (number + 1). In both groups, the mean number of hot flashes was significantly decreased, but no difference was observed.

The mean number of hot flashes before treatment was about 1.7 times/day in the kamishoyosan group and 1.6 times/day in the placebo group, respectively. The mean number of hot flashes was calculated by a mixed effect model using values described in patient diaries and was significantly decreased in both groups (Figure 2).

**Figure 3:**
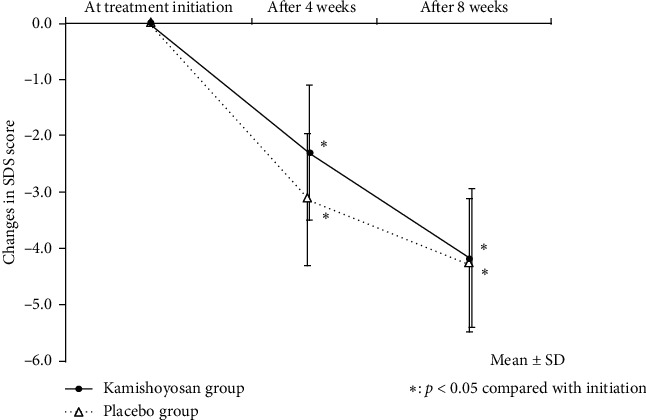
Changes in the SDS score. SDS scores decreased with treatment in both kamishoyosan and placebo groups, but no difference was observed between the two groups. The *y*-axis shows the decrease in the SDS score relative to the pretreatment score.

SDS scores before initiation of treatment were 43.7 (standard deviation (SD) 7.8) in the kamishoyosan group and 44.2 (SD 7.8) in the placebo group. We did not find differences between the groups. SDS scores after 4 and 8 weeks of treatment were 42.5 (SD 8.1) and 40.6 (SD 8.8) in the kamishoyosan group and 40.4 (SD 8.4) and 39.3 (SD 8.4) in the placebo group, respectively. In both groups, these scores were significantly decreased compared to before treatment (*P* < 0.001). However, we did not find differences between the groups in terms of a decrease in SDS score relative to baseline at each assessed time point (Figure 3).

To evaluate how depression status affects kamishoyosan's effect, we conducted reexaminations by dividing subjects into two groups according to pretreatment SDS scores, mild depressive ones with SDS scores ≥44 and not depressive <44. Changes in the SDS score after initiation of treatment were, however, not related to the level of pretreatment SDS score, and indeed, the same pattern of changes as compared to the placebo group was observed. Similarly, we examined the relationship between the effects of kamishoyosan and background factors such as age, menstrual status, and presence or absence of hot flashes. However, in all cases, we did not find differences between the groups (data not shown).

**Figure 4:**
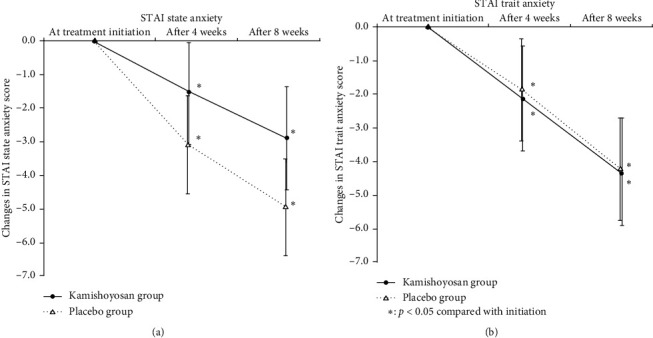
(a, b) Changes in STAI state anxiety and trait anxiety scores. The *y*-axis shows changes relative to the score at treatment initiation, which is set to 0. Both state anxiety and trait anxiety scores showed a significant decrease after the initiation of treatment, but no difference was observed between the groups.

The STAI assessment before initiation of treatment revealed state anxiety and trait anxiety scores of 44.3 (SD 1.1) and 48.8 (SD 1.2), respectively, in the kamishoyosan group, and 47.0 (SD 1.1) and 48.6 (SD 1.1) in the placebo group, and we did not find differences between the two groups. Compared to pretreatment scores, both state anxiety and trait anxiety scores were significantly decreased at 4 and 8 weeks after initiation of treatment in both groups. Also, we did not find differences between the two groups (Figure 4).

**Figure 5:**
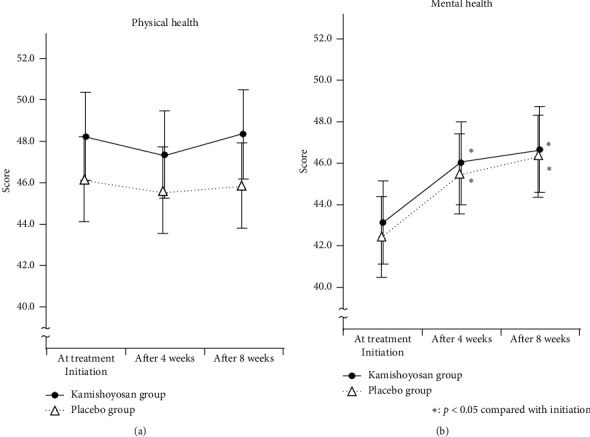
Changes in SF-36 physical health and mental health scores. (a) Physical health. (b) Mental health.

In a health related QOL analysis, kamishoyosan improves mental health scores in SF-36 but not physical health scores (Figure 5). In eight subscales of SF-36, treatment with kamishoyosan led to significant improvements in all scales except for physical functioning at the end point. Compared with the placebo, we did not find differences between the groups.

### 3.4. Adverse Events and Safety

Adverse events were noted in 15 subjects (18 events) in the kamishoyosan group and 7 subjects (8 events) in the placebo group, and 11 subjects (14 events) in the kamishoyosan group and 2 subjects (3 events) in the placebo group could not be denied a causal link to the treatment. In the kamishoyosan group, the serious adverse event was anxiety symptoms in one subject. However, other 7 gastrointestinal complaints, 2 vertigo, 2 articular pain, one palpitation, and one common cold, were not serious. On the other hand, eruption, constipation, and gastric discomfort were found, but not serious, in the placebo group. No subject dropped out due to adverse events.

## 4. Discussion

This study is the first report on the use of the double-blind methodology to verify the effects of Kampo therapy on vasomotor and neuropsychiatric symptoms in menopausal women. Menopausal symptoms are characterized by a wide range of pathologic manifestations and diverse underlying factors. Among factors of menopausal symptoms, decreased ovarian function causes hot flash, sweating, and insomnia [17]. Additionally, neuropsychiatric symptoms are referred to as indefinite complaints, and since these symptoms are not necessarily related to estrogen deficiency, estrogen administration has limited efficacy [18]. For these reasons, psychotropic drugs and Kampo therapy are recommended for treating neuropsychiatric symptoms of menopause.

Kampo therapy is characteristic in that few adverse drug reactions are observed, and that it is well accepted by patients. Many studies have reported on its significance and effects on menopausal symptoms [4, 9, 19]. However, these reports are based on results of open, not randomized trials with no controls. Indeed, only a few reports have scientifically verified whether Kampo therapy is actually effective in treating menopausal symptoms. In particular, menopausal symptoms are closely correlated with the dysfunction of the autonomic nervous system and are thus known to be susceptible to the placebo effect [20]. Thus, in order to verify the effectiveness of Kampo therapy, it is essential to conduct a study under conditions in which biases due to this placebo effect are removed to the extent possible.

Several Kampo medicines are indicated for menopausal symptoms in Japan. In this study, we selected kamishoyosan for the following reasons. Kamishoyosan contains herbal medicines having sedative effects on the central nervous system, such as bupleurum root, ginger, rhizomes of atractylodes lancea, and moutan bark. So, it is indicated for menopausal symptoms and is appropriate for women who get tired easily or suffer from hot flashes, stiff shoulder, and neuropsychiatric symptoms such as dysphoric mood and irritability, which are common in Japan [21]. In addition, kamishoyosan is frequently used in obstetrics and gynecology clinics in Japan and thus was considered suitable for use in this study.

The primary objective of the present study was to investigate the efficacy of kamishoyosan. A hot flash is a typical symptom related to estrogen deficiency, and observing its occurrence as well as changes brought about by treatment is useful in determining the effects of kamishoyosan on menopausal symptoms [4]. The numbers of hot flash decreased in the kamishoyosan group, but there was no significant difference between the kamshoyosan and placebo groups. The small number of initial hot flashes may affect this result. However, it was the same result in other Kampo trials with keishibukuryogan in the USA, with women who had more often hot flashes [7].

In patients with menopausal symptoms, psychological symptoms, as well as physical symptoms, interfere with QOL [22]. Kamishoyosan has been clinically administered to women with psychosomatic symptoms such as irritation, fatigue, anxiety, and depression. To evaluate its effects on these symptoms, the SDS, STAI, and SF-36 were employed. The SDS, STAI, and SF-36 have been used widely to evaluate depression, anxiety, and QOL, respectively, even in the field of menopause [23–25]. While we observed significant improvements of symptoms with the initiation of treatment in all evaluated items, there were no significant differences between the kamishoyosan and placebo groups. With regard to the SDS, scores in kamishoyosan group decreased significantly from baseline to 8 weeks, but there was no significant difference compared with the placebo group. We repeated the assessment by dividing subjects into two groups according to SDS scores of ≥44 and <44, considering the possibility that the mild levels of symptoms at the time of the clinic visit might have prevented kamishoyosan from fully exerting its effects. However, the results were similar. We also reanalyzed the data by stratifying subjects according to background factors such as age, presence or absence of menstruation, and presence and absence of hot flashes, but no significant associations were observed between changes in SDS, STAI, and SF-36 (including subscales) and each factor (data not shown).

There were some limitations of this study. The present study did not consider so-called “patterns” as they are used in the context of Kampo medicine. This was because the “pattern” evaluation is strongly influenced by the subjectivity of the attending physician and is not suitable for a multicenter study. Moreover, many women who complain of menopausal symptoms are considered to belong to the “medium pattern,” which likely matches the pattern of kamishoyosan. Furthermore, since the present study used a placebo drug, the distribution of patterns was considered equivalent. So, the influence of patterns, if any, would have been diminished.

The findings of the present study reassured the safety of kamishoyosan, but did not prove the usefulness of kamishoyosan as a therapeutic drug for patients diagnosed with hot flashes. Nevertheless, our results do not necessarily rule out the effects of kamishoyosan on a variety of symptoms experienced by women during menopause. It is important to note that our evaluations did not assess the direct effects of kamishoyosan on the various symptoms of menopause. Rather, we comprehensively evaluated the secondary symptoms that develop due to the presence of those symptoms. Previous open trials found that kamishoyosan was effective in treating mental and neurological symptoms such as dysphoric mood and irritability, and indeed, kamishoyosan has been shown to be effective in relieving symptoms such as anxiety, irritability, insomnia, dizziness, and headache [4]. Women during menopause complain of a variety of symptoms, and the effects of these symptoms depend on the period since onset. This viewpoint was lacked in this study. Additionally, the limitation of the present study is that this temporal factor was not taken into consideration. Another issue was that, since the evaluation of menopausal symptoms is subjective in nature, it is difficult to measure the severity of symptoms objectively. Moreover, menopausal symptoms are indefinite complaints that often change from day to day and can vary considerably with a sense of relief from communicating with a physician or taking medication. Third, we observed the positive effects of placebo in all indexes. In other words, we reconfirmed the susceptibility of menopausal symptoms to the placebo effect. It is well known that treatments for menopausal symptoms show large placebo effects. In another study with equal soy isoflavone metabolite, the placebo effect seems to be about 30–35% [26]. Especially, psychological symptoms tend to be influenced by placebo effects, and, in this study, improvement of mental health measured by SF-36 in the placebo group is similar to that in the kamishoyosan group. Therefore, in order to clarify the true effect of kamishoyosan, it will be necessary to conduct research with a new study design to remove these biases.

In conclusion, this first placebo-controlled double-blind randomized trial with kamishoyosan demonstrated that it was safe and had some effects on climacteric symptoms, but not significant compared with placebo. Some problems, such as placebo effects, in the study of Kampo therapy for menopausal symptoms, were revealed.

## Figures and Tables

**Table 1:** Patient characteristics.

	Kamishoyosan group (*N* = 101)	Placebo group (*N* = 104)
Age (years)	50.6 (4.3)	50.8 (4.3)
Height (cm)	158.9 (4.8)	157.9 (4.7)
Body weight (kg)	56.1 (9.3)	56.4 (9.9)
Systolic blood pressure (mmHg)	127.0 (18.8)	127.2 (19.0)
Diastolic blood pressure (mmHg)	79.0 (13.4)	80.0 (13.1)
Postmenopausal (number of patients (%))	64.2	67.3
Number of births	1.7 (1.2)	1.3 (1.1)
Drinking habit (%)	59.4	51.0
Smoking habit (%)	22.2	18.4
Previous treatment for climacteric symptoms with medication (%)	14.9	10.8
Past medical history (%)	56.4	53.8
Complicating disease (%)	50.5	55.8
Concomitant medication (%)	40.6	47.1
Pretreatment SDS	43.7 (7.8)	44.2 (7.8)
Pretreatment SDS median	44	44
Pretreatment SDS range	27–59	28–61

Number inside parentheses indicate mean (standard deviation)

## Data Availability

The data used to support the finding of this study are available from the corresponding author upon request.
